# Remote Results of Arthrotomies of the Knee

**Published:** 1918-12

**Authors:** 


***Remote Results of Arthrotomies of the Knee.*** By MM. Mouchet and Pamart. *Bulletin de la Société de Chirurgie de Paris*, April 30, 1918.

The authors have studied 54 cases of arthrotomies of the knee following wounds occasioned in most cases by shell fragments : 39 per cent. of these have had good results; 25 per cent., fair results; 35 per cent., bad results, i. e., more or less complete ankylosis of the knee.

These cases are about a year old, and have been treated by the latest and most perfect methods of healing joint wounds : early interventions, removal of the projectile, and very thorough cleansing of the joint.

The authors conclude, from the cases they studied, that the results of the arthrotomies of the knee are on the whole satisfactory. They think they might be improved if the patient were not compelled to remain so long immobilized.

They consider that the great arthrotomy in U form gives the worst results and is practised much too often. They think it ought to be strictly reserved for the very serious wounds of the knee, when a large space is needed for the operation.

